# Green Composites Based on PLA and Agricultural or Marine Waste Prepared by FDM

**DOI:** 10.3390/polym13091361

**Published:** 2021-04-21

**Authors:** Roberto Scaffaro, Andrea Maio, Emmanuel Fortunato Gulino, Giuseppe Alaimo, Marco Morreale

**Affiliations:** 1 Research Unit INSTM, Department of Engineering, University of Palermo, Viale delle Scienze, 90128 Palermo, Italy; emmanuelfortunato.gulino@unipa.it (E.F.G.); giuseppe.alaimo07@unipa.it (G.A.); 2 Faculty of Engineering and Architecture, Kore University of Enna, Cittadella Universitaria, 94100 Enna, Italy

**Keywords:** *Posidonia oceanica*, *Opuntia ficus indica*, polylactic acid, additive manufacturing, 3D printing, aspect ratio, biocomposites, degradation, mechanical properties, water contact angle

## Abstract

Three dimensional-printability of green composites is recently growing in importance and interest, especially in the view of feasibility to valorize agricultural and marine waste to attain green fillers capable of reducing bioplastic costs, without compromising their processability and performance from an environmental and mechanical standpoint. In this work, two lignocellulosic fillers, obtained from *Opuntia ficus indica* and *Posidonia oceanica*, were added to PLA and processed by FDM. Among the 3D printed biocomposites investigated, slight differences could be found in terms of PLA molecular weight and filler aspect ratio. It was shown that it is possible to replace up to 20% of bioplastic with low cost and ecofriendly natural fillers, without significantly modifying the processability and the mechanical performance of the neat matrix; at the same time, an increase of surface hydrophilicity was found, with possible positive influence on the biodegradability of such materials after disposal.

## 1. Introduction

Environmental issues related to plastic waste, along with the high cost of currently developed bioplastics, stimulated the research on green composites, aiming at fulfilling the demand for materials that possess high sustainability from an economic and ecological standpoint [1,2,3,4,5]. Green composites are typically obtained by combining biodegradable and/or bioderived polymer matrices (although traditional, non-biodegradable polymers may be used, but with obvious disadvantages in terms of environmental impact) with natural-organic fibers/particles, either as mere fillers or reinforcements. Polymer matrices can be thermoplastics or thermosets and, in general, thermoplastics are preferred also due to their easier processability and recyclability. Besides the environmental and cost advantages, green composites are often appreciated by customers because of their distinctive aesthetic properties and more “natural” look, even though some mechanical properties can be sometimes reduced in comparison to neat polymers [3].

It is known that compression, extrusion and injection molding are the main used technologies, for the production thermoplastic based green composites [6]. However, it is of great importance to investigate possible alternative ways for the processing of green composites. Over the last years, for instance, a dramatically increasing interest has risen towards the fused deposition modeling (FDM) technique in several applications. Thus, the possible application of this technique to green composites manufacturing has led to several studies available in the literature, mainly regarding the optimization of the process parameters in the case of neat polymers. Three dimensional printing based on FDM additive process allows to set different parameters of printing, which lead to direct and specific consequences in the mechanical properties of the final product.

Popescu et al. [7], for instance, highlighted the importance of a proper choice of the process parameters and their effects on the mechanical properties of the obtained composites. In this review study, a detailed discussion was provided about the slicing parameters (layer thickness and height, nozzle diameter, deposition flow and speed, infill rate, external pattern and internal pattern, number of perimeters, air gaps, etc.), building orientation (horizontal, vertical, or side orientation) and temperature conditions (extrusion, bed and room temperature).

Several experimental studies focused on the effects on the properties of the manufactured systems, using acrylonitrile-butadiene-styrene copolymer (ABS) [8,9] or polylactic acid (PLA) [10,11,12,13,14].

Interesting is the study on PLA by Hongbin et al. [11], who evaluated the effect of process parameters on the degree of bond in the PLA between the 3D-printed filaments, which is directly correlated to the mechanical properties of the product. They reported an increase of the degree of bonding between filaments as the thickness of the layer decreased, with consequent increase of the tensile resistance. Deposition speed showed the same dependencies of the layer thickness and increasing infill rate led to increasing tensile resistance.

With regard to FDM manufacturing of green composite samples, the filler presence and amount is an additional process variable that leads to further challenges and clearly depends also on the filler type.

Kariz et al. [15], for instance, evaluated the effects of the amount of wood fibers (up to 50%) in 3D-printed samples based on PLA and ABS. They found that the orientation of the wood fibers followed the one of the filament printing/drawing, reducing the density of the filaments. On average, the tensile strength increased up to 10% filler load, while the elastic modulus increased up to 20% filler load, and then both experienced significant reductions on increasing the filler content. This was partly attributed to filament surface morphology, becoming more and more rough and porous, with filler particle agglomerates, which could also lead to clogging during the deposition stage.

Duigou et al. [16] investigated on the properties of 3D-printed PLA filled with continuous flax fibers (cFF). The cFF amount was up to 30.4 *v/v* %. The Young modulus showed dramatic increase in comparison to the unfilled PLA, but the properties in the transverse direction were lower than those obtainable on the same systems, prepared via compression molding.

The possibility of using lignin, the main component of lignocellulosic fibers, was also investigated. Gorbodil et al. [17] prepared PLA composites filled with commercial lignin, finding that the presence of lignin hinders the hydrolytic degradation of PLA, while the crystallinity decreased, leading also to a reduction in the elastic modulus and tensile strength, while the elongation at break increased. Gao et al. [18] studied the effects of different types of lignin, extracted from corn harvesting residues and selected on the basis of the purity degree. On average, all the obtained PLA composites showed significant improvements of the elastic modulus, while the breaking properties worsened on increasing the lignin amount.

Among the several alternative lignocellulosic fillers that can be considered for the manufacturing of green composites, with the aim of reducing the environmental impact and optimizing the use of natural resources, (both renewable and not renewable) *Opuntia ficus indica* (OFI) and *Posidonia oceanica* leaves (POL) have recently gained attention. The former is abundant in the Central America and Mediterranean regions, and the leaves are a typical agricultural waste, which can be exploited by extracting several substances and residues (such as, for instance, lignocellulosic fibers). The latter is a seaweed, very abundant in the Mediterranean region and constituting a significant trouble because of its tendency to accumulate on the seaside, with detrimental effects on beach quality and forcing local authorities to bear additional costs for collecting and disposal.

Both of them may be, therefore, of interest in the view of utilization in the manufacturing of green composites.

Greco et al. [19], for instance, prepared PLA-based biocomposites by compression molding, and compared their properties upon using flax or OFI fibers, finding interesting results in terms of tensile properties.

Scaffaro et al. [20] investigated on the structure-properties relationships of PLA-OFI biocomposites, selecting two different size classes (<150 μm and 150 μm–300 μm), two different filler amounts (10% and 20%) and adopting compression molding as the manufacturing technique. On average, they found an increase in the elastic modulus, while the deformability decreased on increasing the filler content; the filler–matrix adhesion was better in the 150–300 μm range, due to the polymer entering the lymphatic vessels of the fibers.

The same group [21] prepared PLA/PO composites via compression molding, selecting two different PO percentages (10 and 20 wt %) and granulometric classes (<150 μm and 150–300 μm). It was found an increase of tensile and flexural modulus, while elongation at break and tensile strength experienced some reduction, less significant in the 10% filled sample and attributed also to a better dispersion and matrix–filler interaction.

Benito-González et al. [22] used cellulose extracted from PO to improve the properties of polymer-based films for packaging applications. The matrix was a corn starch-based biopolymer, and the filler amount was up to 10 wt %. The results were encouraging since significant improvements of the elastic modulus and of the barrier properties were found.

It is clear from this brief literature review, that there are not systematic studies available on the FDM and subsequent characterization of PLA/OFI and PLA/POL biocomposites. This would be of significant interest in order obtaining 3D-printed objects with lower environmental impact and reduced cost, in comparison to neat PLA.

In this work, therefore, we assessed the suitability of the PLA/OFI and PLA/POL biocomposites to the FDM process. More in details, suitable filaments were produced via extrusion and then the 3D-printing process variables were chosen and the obtained samples properties (mechanical, rheological/structural, morphological and their wettability), before and after FDM, were investigated.

## 2. Materials and Methods

### 2.1. Materials

**Figure 1:**
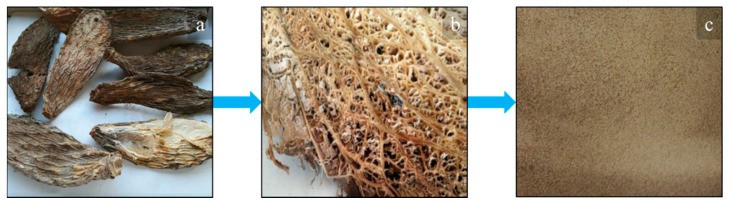
Raw OFI cladodes before (**a**) and after (**b**) washing and after chopping (**c**).

**Figure 2:**
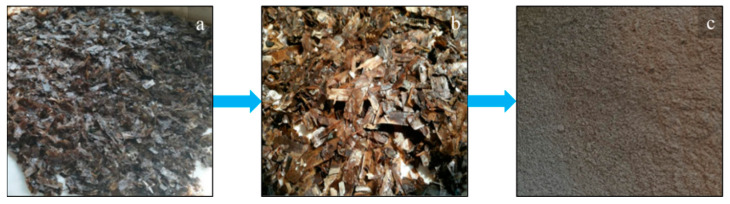
Raw POL before (**a**) and after (**b**) washing and after chopping (**c**).

The PLA used in this work was a 2002D extrusion grade, produced by Natureworks (USA). It is an amorphous sample, having 4% content of D-lactic acid monomer, *ρ* = 1.24 g/cm^3^, MFI = 6 g/10 min, Mn = 113,300 and Mw = 181,600, processable at a temperature above 155 °C. With regard to the natural organic fillers, the cladodes from *Opuntia ficus indica* (OFI) were supplied by Bio Ecopuntia (Italy), while scraps of *Posidonia oceanica* leaves (POL) were collected on Palermo coast, Italy. The obtained raw materials (Figure 1a and Figure 2a) were then thoroughly washed (Figure 1b and Figure 2b) and chopped (Figure 1c and Figure 2c) before further processing. According to previous studies, both fillers display a porous structure, due to the presence of hexagonally shaped, vascular empty channels, called lumens [20,23]. The dimensions of these latter were found to be more regular in the case of POL, with each pore showing area approximately equal to 50–60 μm^2^, and much irregular in the case of OFI, whose single pores showed areas varying from a few tens of μm^2^ up to 2000 μm^2^. Due to the high porosity levels of both fillers, a discrepancy was found between the tensile properties of raw whole fibers and the calculated bulk modulus. In details, OFI raw fibers have an elastic modulus equal to 3.5 (±0.6) GPa and 60% porosity, which corresponds to a bulk modulus of 20 GPa, whereas POL fibers display 0.85 GPa with 73% porosity, thus possessing bulk modulus of 10 GPa. The chemical composition of the two lignocellulosic fillers reveals high content of polysaccharides, ashes and other extractives [24,25]. In more detail, POL contains practically the same amounts of cellulose (31%) and lignin (29%), beyond hemicellulose (26%) and ashes (10%), whereas OFI cladodes display lower contents of cellulose (20%) and especially lignin (only 3%), with higher amounts of hemicellulose (48%), ashes (20%) and other components, such as fat and wax (7%) [24,25].

### 2.2. Preparation

The first step was focused on obtaining OFI and POL particles suitable to be used in the 3D printer without nozzle clogging. Indeed, such systems proved to show mechanical performance increasing with filler size, which was found to be beneficial for several reasons [20,23]. First, it was assessed that the larger the filler size, the higher the PLA capability to enter the empty lumens of such porous fillers, thus leading to more compact structures [20,23]. Second, the aspect ratio of both fillers was found to decrease upon decreasing granulometry, since during grinding, failure preferentially occurs along fiber axis [20,23]. Third, small sized lignocellulosic fillers demonstrated strong prodegradant activity toward PLA [20,23]. Furthermore, the smaller the size the lower the yield during grinding plus sieving procedures, with obvious disadvantages in terms of costs and time. Therefore, OFI and POL were grinded for 2–3 min and then sieved in a 200-mesh sieve, aiming to select fillers having a size below 75 µm.

The obtained OFI and POL flours were conditioned in a vacuum oven at 90 °C overnight, before each processing operation.

In detail, neat PLA and PLA-based biocomposites containing 10 and 20 wt % OFI, (OFI10 and OFI20 respectively) and POL (POL10 and POL20, respectively), were prepared in a Haake (Germany) Polylab single-screw extruder (L/D = 25; D = 19.05 mm), operating at 40 rpm screw speed and 160–180–200–210 °C temperature profile. The filler amounts were chosen on the basis of previous studies [20,23]. The extrudates were drawn with the help of a conveyor belt system (take-up speed = 5.5 m/min), to obtain filaments with a steady 1.75 mm diameter, suitable to the following 3D printing step.

**Figure 3:**
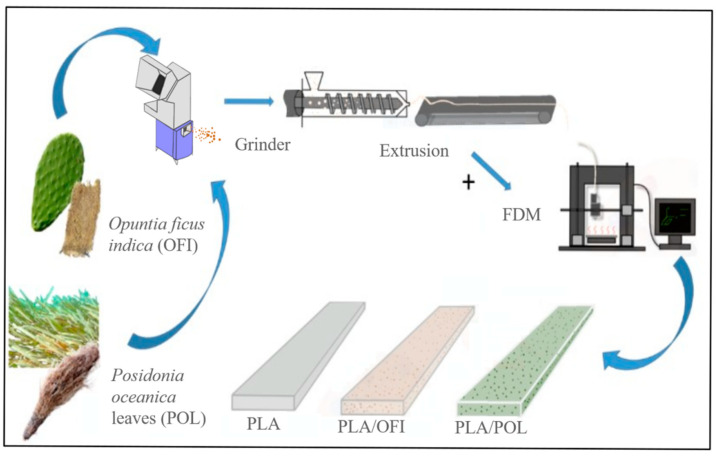
Schematic representation of the process.

The entire process is visually summarized in Figure 3.

### 2.3. FDM of the Biocomposites

Design of the samples to be obtained via FDM was performed with the help of the CAD Solid Edge 2019^®^ software. STL files produced were elaborated on Simplify3D^®^ software to obtain the gcode files. More in detail, for each biocomposite system, the following specimens were designed: 60 mm × 10 mm × 2 mm for tensile tests, 40 mm × 13 mm × 2 mm for flexural tests and 80 mm × 10 mm × 2 mm for impact tests. FDM process was carried out using a Sharebot Next Generation (Italy) 3D printer. The FDM parameters are reported in Table 1. The nozzle and bed temperature were chosen after some trials, aiming to avoid nozzle obstructions and to obtain good adhesion of the sample on the bed, with good dimensional stability. The other parameters were chosen based on the scientific literature. In particular, a rectilinear infill pattern with a 0° raster angle was chosen in order to optimize the tensile properties (especially, the rigidity); 80% infill rate and 45 mm/s printing speed were chosen as a compromise solution between better properties and higher production rate; three perimeter shells also on the basis of studies on similar systems using FDM 3D-printing [8,9,10].

**Figure 4:**
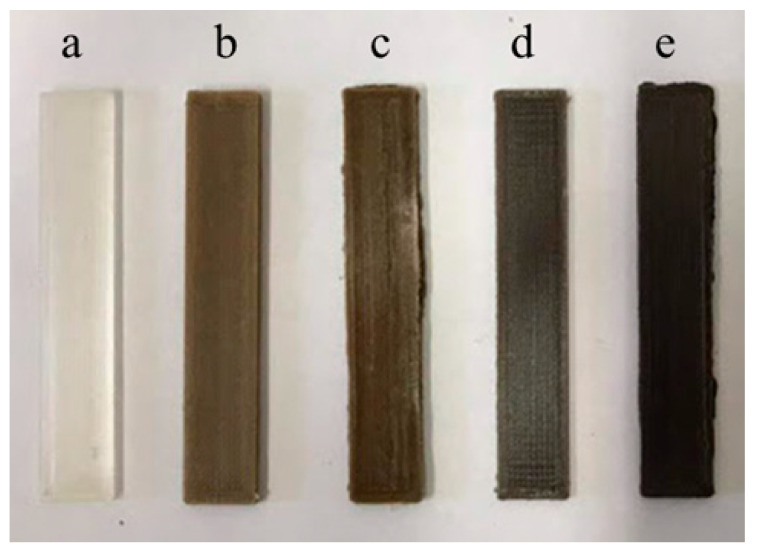
Representative specimens for tensile tests: PLA (**a**), OFI10 (**b**), OFI20 (**c**), POL10 (**d**) and POL20 (**e**).

The obtained 3D-printed samples and their codes are summarized in Table 2, while some representative samples are shown on Figure 4.

### 2.4. Morphological Characterization

The morphology of starting materials and resulting biocomposites was analyzed by scanning electron microscopy (SEM), performed by means of a PhenomPro X microscope onto gold-sputtered (Sputtering Scancoat Six, Edwards) samples to avoid electrostatic discharge during the test.

### 2.5. Aspect Ratio of the Fillers via Image Analysis

The geometrical features of the particles, i.e., the resulting aspect ratios (A_r_), were measured by using an open-source software, Image J by NIH Image (JAVA-based image analysis software) both before and after processing to analyze the effect of the filler size on biocomposites properties. OFI aspect ratio was measured through length (L) and diameter (D), and POL aspect ratio through highest length and thickness.

### 2.6. Intraphase Calculation via Density Measurements

For each system, the aliquot of microparticle lumens entered by PLA (i.e., the percentage of intraphase) was calculated by helium pycnometer, according to the following equation:

(1) $$intraphase\;\left(\%\right)=\frac{\rho_{c}-\rho_{empty}}{\rho_{filled}-\rho_{empty}}$$

where $\rho_{c}$ is the real density of the given composite, calculated by He pycnometer, while $\rho_{filled}$ and $\rho_{empty}$ are, respectively, the theoretical density values that the composite would assume in the limiting cases of: (i) absence of voids, that is: particle lumens totally entered by PLA (intraphase = 100%) and (ii): totally empty lumens (intraphase = 0%). These latter parameters were assessed by the Equations (2) and (3):

(2) $$\rho_{filled}=\rho_{f}\Phi_{f}+\rho_{PLA}\Phi_{PLA}$$

(3) $$\rho_{empty}=\rho_{a,f}\Phi_{a,f}+\rho_{PLA}\Phi_{PLA}$$

where $\rho_{f}$ is the real density of the filler, measured by pycnometer, and $\Phi_{f}$ its volumetric fraction in the composite by considering this density value, while $\rho_{a,f}$ and $\Phi_{a,f}$ respectively represent the apparent density of the filler and the ensuing volumetric fraction under the hypothesis that none of its lumens is entered by the matrix.

### 2.7. Mechanical Characterization

Tensile properties (elastic modulus, E, tensile strength, TS, and elongation at break, EB) were measured using an Instron mod. A 3365 (USA) dynamometer on the above described rectangular specimens (60 × 10 × 2 mm^3^), by setting a double crosshead speed (1 mm/min for the first two minutes, and then 50 mm/min up to specimen break). The tests were carried out at T = 25 °C and 50% RH onto at least seven specimens for each sample.

Flexural tests in three-point bending mode were performed onto 40 × 13 × 2 mm^3^ specimens, according to the ASTM D790 standard, by using the same apparatus under the same environmental conditions, aiming to measure the flexural modulus (FM) and flexural strength (FS).

Impact tests in the Charpy mode were performed on the 80 × 10 × 2 mm^3^ specimens, using a pendulum model 9050 by CEAST (Italy), according to the ASTM D6110 standard, to evaluate impact strength (IS).

### 2.8. Water Contact Angle (WCA) Measurements

Surface wettability of the obtained samples was determined through WCA testing, performed using a First Ten Angstroms (UK) FTA 1000 equipment on at least three specimens per material. Of distilled water 4 μL dropped, at room temperature, on the samples by an automatic liquid drop dosing system. Water dropped images were taken after 20 s; at least four spots of each sample were tested and the average values of WCA where then calculated.

### 2.9. Molecular Weight Assessment via Intrinsic Viscosity

Intrinsic viscosity, (η), was measured using a Lauda (Germany) Proline PV 15 apparatus equipped with a Ubbelohde capillary viscometer (K = 0.009676) immersed in an oil bath at T = 30 °C. The samples were first dissolved in chloroform (CF) for 3 h at room temperature under agitation in a magnetic stirrer, and then filtered with the help of a water vacuum pump. Thereafter, the recovered polymer was exsiccated and then dissolved again in CF under agitation for 1 h. The obtained solution at 0.13% (wt/wt) concentration was then used for the flow time measurements in the capillary. The intrinsic viscosity values were then calculated using the Solomon-Ciuta equation [26]:

(4) $$\left[\eta \right]=\frac{\surd 2\left(\eta_{s}-{\ln\;\eta }_{r}\right)}{C}$$

where *C* is the concentration of the polymer solution, η*_s_* and η*_r_* are the specific and the relative viscosity, respectively. The viscosity of each sample solution was obtained by the average of three flow measurements.

The average viscosimetric molecular weight, *M*, was calculated using the Mark–Houwink equation:

(5) $$\left[\eta \right]=KM^{\alpha }$$

where *K* and *α* are dependent on the specific polymer-solvent system, and in this case (PLA in ClCH_3_ at 30 °C) they are: *K* = 0.0153 mL/g and *α* = 0.759 [27].

## 3. Results and Discussion

**Figure 5:**
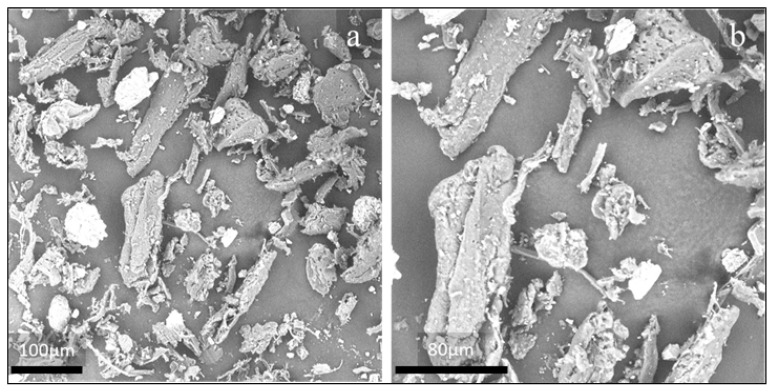
SEM micrographs of OFI fibers before processing, at 500× (**a**) and 1000× (**b**) magnification.

**Figure 6:**
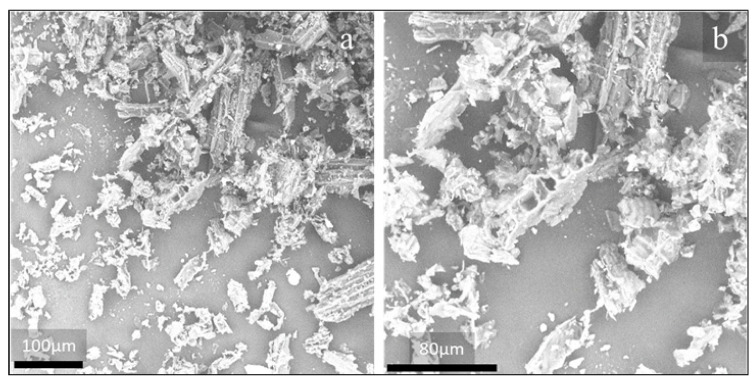
SEM micrographs of POL fibers before processing, at 500× (**a**) and 1000× (**b**) magnification.

First, size distribution of the natural organic fillers after sieving was analyzed by SEM, and relevant micrographs are shown in Figure 5a,b and Figure 6a,b for OFI and POL fibers, respectively.

**Figure 7:**
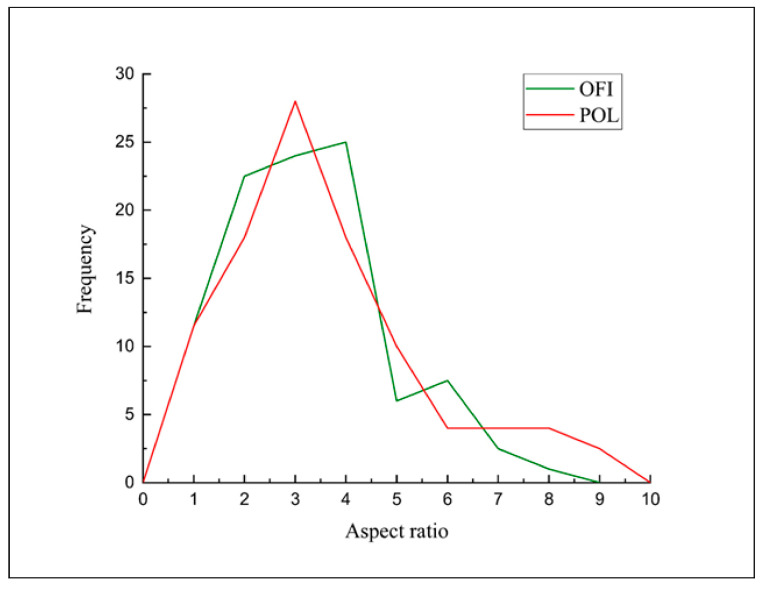
Size distribution of POL and OFI fillers, before processing.

In both cases, it is easy to observe the elongated shape of the particles, kept even after sieving. Therefore, aspect ratio distribution was taken and reported in Figure 7.

It can be easily noted that the prevalence of particle size is in the range 2–4 of A_r_ for both fillers, therefore the influence of possibly different size distributions before processing is relatively negligible.

**Figure 8:**
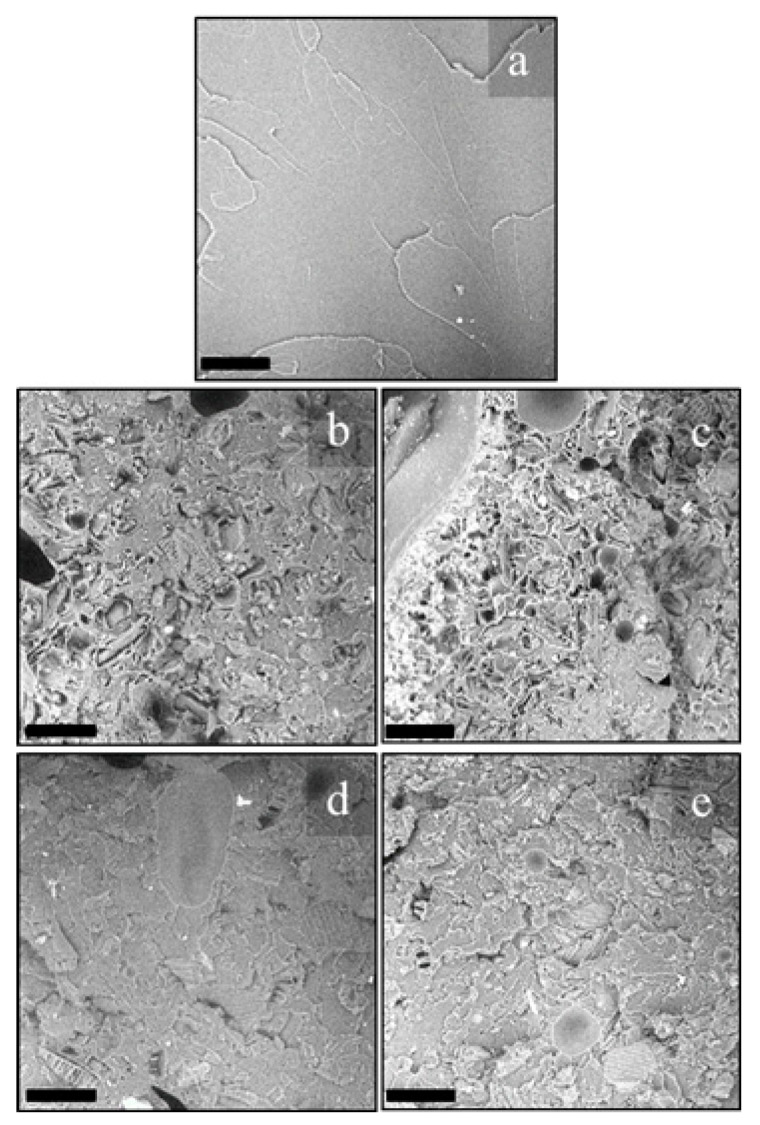
SEM micrographs of cryofractured cross-sections of PLA (**a**), OFI10 (**b**), OFI20 (**c**), POL10 (**d**) and POL20 (**e**) extruded filaments; scale bar 100 μm.

**Figure 9:**
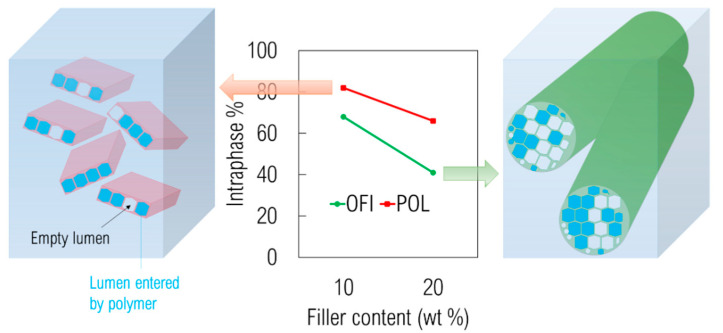
Intraphase plotted as a function of filler content for systems containing OFI and POL, together with pictorial representations of empty and filled lumens in POL10 (**left**) and OFI20 (**right**).

Morphological analysis was also performed on transverse fracture surfaces of the filaments obtained after extrusion, for evaluating the dispersion and/or possible particle agglomerates in the extruded filaments. Figure 8 shows the fracture surfaces of PLA, OFI10, OFI20, POL10 and POL20 filaments. In the PLA-OFI system, the presence of the filler leads to few agglomeration phenomena, and high levels of voids due to the presence of lumens not entered by the polymer; these phenomena increase on increasing the filler content. In our previous work, we had already envisaged that the capability of PLA to enter the empty channels of OFI proved to decrease upon decreasing filler size. This feature, that we introduced for the first time and called “intraphase” is a crucial factor to investigate the structure–property relationship of all those biocomposites containing porous fillers. In fact, high intraphase degrees determine higher contact area between filler and matrix and, of course, a lower level of voids with positive repercussions on the ultimate mechanical performance of biocomposites. In fact, in the ideal case of intraphase close to 100%, elastic modulus of fillers would tend to their bulk modulus. Similarly, it can be easily demonstrated that intraphase degrees close to 0 would lead to levels of voids comparable with the porosity of raw fillers, with negative repercussions on mechanical properties. In the case of PLA-POL biocomposites, the filler dispersion proved to be more uniform and, especially, an extremely lower level of voids was found, with most of the lumens being penetrated by polymer chains. A quantitative assessment of intraphase for such samples was performed via pycnometry, according to the procedure described in our previous works [20,23] (see Equations (1)–(3)) and the results are provided in Figure 9, together with the pictorial representations of POL10 and OFI20, respectively on the left and on the right of the plot. As one can see, the quantitative measurements herein reported strongly confirm what envisaged by morphological analysis. The capability of PLA to enter the empty channels proved to be different, depending on the type of lignocellulosic filler, with POL being more suitable than OFI. In both cases, however, the level of voids tends to increase at higher filler loading.

However, after FDM and production of the 3D printed systems, the most straightforward way to evaluate the quality of the obtained samples, relies on the measurement of the main mechanical properties. Tensile, flexural and impact (in Charpy mode) properties of the samples are reported in Table 3, Table 4 and Table 5, respectively.

With regard to the tensile properties, the presence of the fillers led to a significant reduction in the breaking properties (TS and EB) at high filler content, with decrements up to 50% in the case of OFI20, while 10% loaded composites displayed negligible losses. The elastic modulus keeps substantially steady, in agreement with other studies on 3D printed biocomposites [15]. Similar considerations can be drawn with concern to the flexural properties, although the decrements observed in terms of moduli are more significant, going from 11% (POL10) to 36% (OFI20).

As far as the impact properties are concerned, on the other hand, there is a beneficial effect of the filler presence up to 10 wt %, with POL10 and OFI10 displaying relative increments of 14% and 20%, respectively; while a sharp change in this trend occurs at higher amounts, in agreement with other studies on similar systems [18].

An overview of relative variations of the main mechanical properties observed in biocomposites is provided in Table 6.

Taken together, these outcomes point out that the best performance was showed by POL10, whereas OFI20 offered the worst performance. Notably, these results were in full agreement with the trend observed for intraphase percentage (see again Figure 9), thus suggesting that the different ability of PLA in penetrating the filler voids could have played a key-role in determining the ultimate performance of such materials.

**Figure 10:**
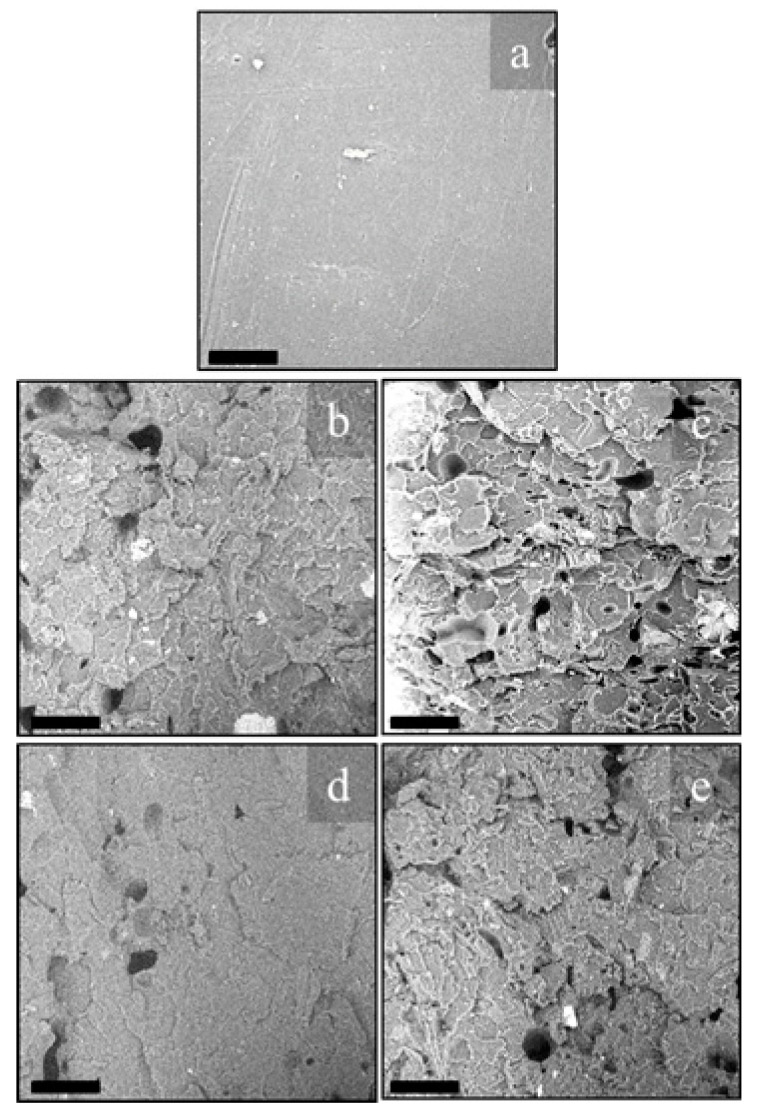
Fracture surfaces after tensile tests of PLA (**a**), OFI10 (**b**), OFI20 (**c**), POL10 (**d**) and POL20 (**e**) 3D-printed samples; scale bar 100 μm.

In order to better explain these results, SEM micrographs of the fracture surfaces (after tensile tests) of the 3D printed specimens were taken and reported in Figure 10.

**Figure 11:**
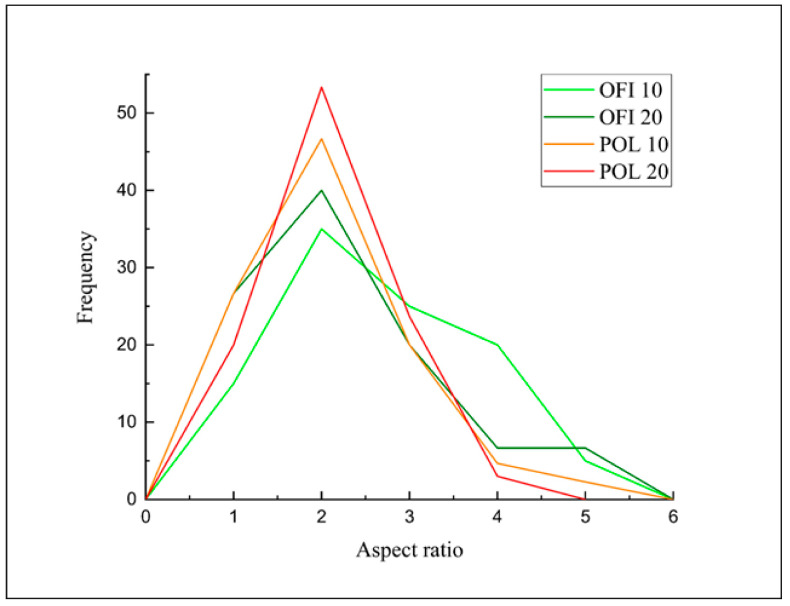
Size distribution of filler particles in OFI10, OFI20, POL10 and POL20 3D-printed samples.

As expected, even from the fracture surfaces of the extruded filaments, higher filler contents lead to rougher fracture surfaces, with the presence of few agglomerates and, mostly, voids (related to fillers’ porosity) and cracks (obviously related to stress concentration phenomena) [28]. The two fillers share these trends, albeit in POL20 it was possible to see a fair amount of lumens totally filled by the polymer, whereas OFI20 exhibited the highest concentration of voids. Even in this case, POL10 showed the highest compactness, in full agreement with the outcomes of the intraphase study. The decrements observed in the mechanical properties are further explainable considering that similar breaking of the microfibers during processing occurred, as evidenced by image analysis of the fracture surfaces, which allowed calculating the size distribution reported in Figure 11.

It was calculated that, while the average Ar before processing of OFI microfibers was about 3, and that of POL microfibers was about 2.6, their reductions after processing (i.e., in the 3D-printed samples) were about 34% and 44% for OFI10 and OFI20, respectively, and about 27% and 36% for POL10 and POL20, respectively. Therefore, although fiber breaking was slightly lower in the POL composites in comparison to OFI ones, this was not sufficient to lead to remarkably different mechanical properties of the two biocomposite systems, probably because of similar filler–filler aggregation and polymer–filler interaction patterns.

**Figure 12:**
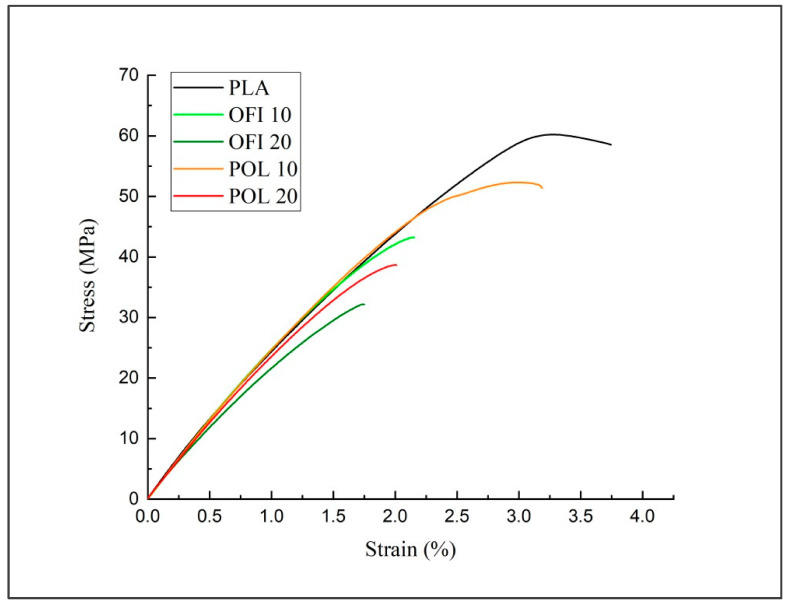
Stress–strain curves of the investigated systems (tensile tests).

**Figure 13:**
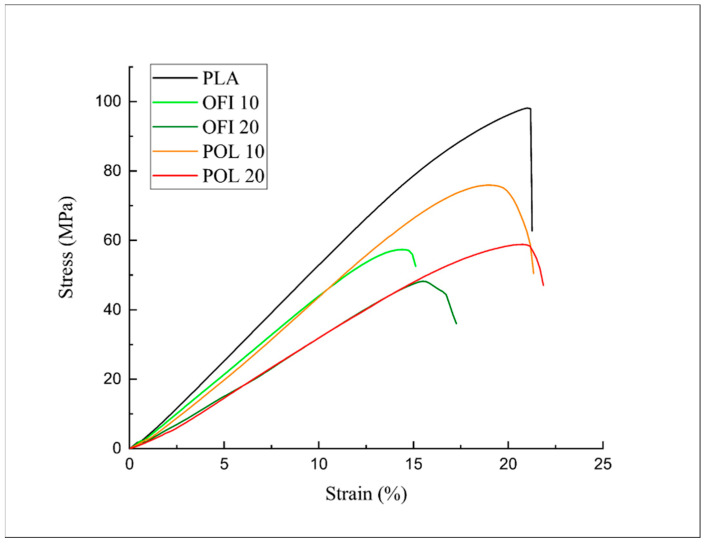
Stress–strain curves of the investigated systems (flexural tests).

The differences in the mechanical response can be further observed from the stress-strain curves in the tensile (Figure 12) and flexural (Figure 13) mode.

It is also relevant to investigate if significant changes in the polymer matrix characteristics occur during processing. This was assessed on the basis of molecular weight modifications, through intrinsic viscosity measurements. The calculated molecular weights for the extruded and the FDM samples are reported in Table 7.

**Figure 14:**
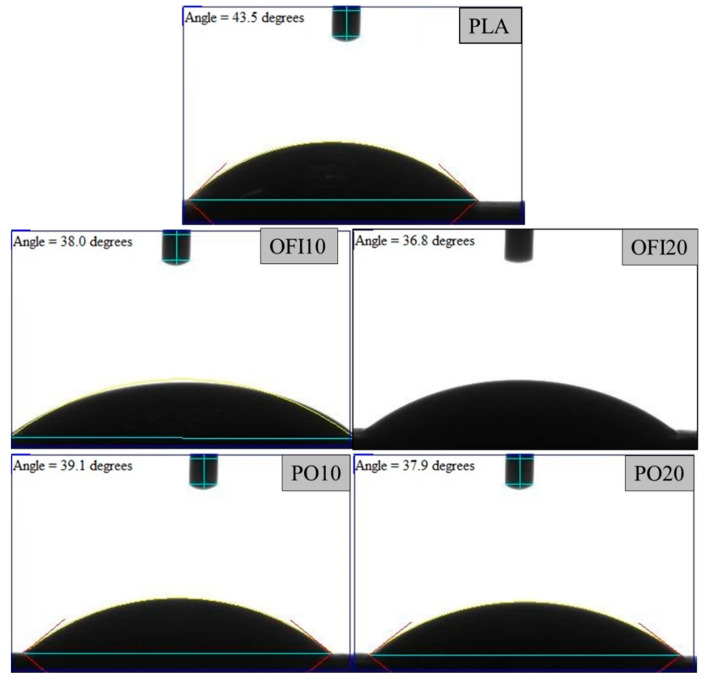
Representative WCAs of 3D printed PLA and related biocomposites.

It can be noticed that due to the double processing step (single-screw extrusion plus 3D printing), the FDM samples had significantly lower values of the molecular weight, with approximately 20% loss in the PLA and 22–25% in the composites. Furthermore, in both series of samples, the reduction increased on increasing the filler content. This can be explained considering that higher filler loadings in similar systems have been demonstrated to lead to higher viscosity [23,29] and thus to higher shear stresses. These, in turn, lead to higher thermomechanical degradation. However, the differences between 10% and 20% filled systems are relatively small in the FDM samples, indicating that the 3D printing process, in the configuration and with the parameters adopted in this work, does not involve particularly high shear stresses. This provides further elements that confirm the effectiveness of the process setup and parameters selection performed in this work. Finally, representative water contact angles (WCAs) of FDM samples are shown in Figure 14, whereas mean values and standard deviations are listed in Table 8.

The main observations that can be drawn in regard to the hydrophilic character of all these systems, especially on increasing the filler content, with WCAs ranging from 43.4 to 36.9 degrees. In the case of neat PLA, a WCA as low as 43.4° could be somehow unexpected, given the typical hydrophobicity of this polyester. However, it can be easily explained considering the significant molecular weight reduction occurred because of the double processing, with ensuing increase of -COOH and -OH end groups [30]. Indeed, this result is consistent with literature data [14]. Further reduction in WCA values was observed in all the biocomposites. These values, moreover, decrease on increasing the filler content. This means that the investigated systems become increasingly hydrophilic, and this can be therefore mainly attributed to the hydrophilic nature of the natural-organic fillers, although being incorporated in the PLA matrix. This result can lead to beneficial effects because it could favor the interaction with water and bacteria and thus increase the overall material degradability, both from a chemical and biological standpoint. In fact, it is well-known that some polyesters, while being fully degradable, often experience extremely slow degradation kinetics because of their hydrophobicity, which hinders water diffusion into the bulk [2,31]. The actual biodegradation kinetics and performance would deserve further investigation and will be the object of a future work. Moreover, among the possible future perspectives, it could be explored the feasibility of such systems in water treatment applications, relying to their porosity and hydrophilicity, as a green and cheap alternative to recently proposed materials [32,33].

## 4. Conclusions

In this work, the feasibility of FDM in the production of green composites was assessed. Two different natural fillers were obtained from marine and agriculture wastes, namely *Posidonia oceanica* leaves (POL) and *Opuntia ficus indica* (OFI) cladodes, ground into flour and then integrated into a PLA matrix at two different loading levels. The outcomes of this research outline that it is possible replacing up to 20% of bioplastic with low cost and ecofriendly natural fillers, without significantly altering the processability and the mechanical properties of the neat polymer. Despite the initial differences in terms of shape, aspect ratio and stiffness, the two fillers behaved in a substantially similar way, with all the mechanical features being mainly governed by loading content and interlayer adhesion during FDM. Indeed, after processing, negligible differences could be found in terms of both PLA molecular weight and filler aspect ratio among the 3D printed biocomposites. Furthermore, biocomposites proved to be more hydrophilic than neat polymer, and this could favor the biodegradability/compostability of the investigated materials after disposal. Of course, stability of matrix towards thermomechanical degradation and filler–matrix interactions might be enhanced by using chain extenders and coupling agents, whether high mechanical performance is needed. Nevertheless, the valorization of natural scraps for the cleaner production of ecofriendly materials with satisfactory mechanical properties and aesthetic features widely appreciated by consumers, could pave the road for the green fabrication of furniture panels, objects, toys and so on, in full compliance with zero-waste and circular economy guidelines.

## Figures and Tables

**Table 1:** FDM process parameters adopted in this work.

FDM Operating Parameter	Value
Nozzle temperature	220 °C
Bed temperature	60 °C
Infill rate	80%
Infill pattern	Rectilinear
Raster angle	0°
Layer thickness	0.1 mm
Extrusion width	0.45 mm
Printing speed	45 mm/s
Perimeter shells	3
Sample Orientation	flat

**Table 2:** 3D printed samples and related codes.

Sample Code	PLA, wt %	Filler, wt %
PLA	100	0
OFI10	90	10
OFI20	80	20
POL10	90	10
POL20	80	20

**Table 3:** Tensile properties of the 3D printed samples.

Sample	Elastic Modulus (MPa)	Tensile Strength (MPa)	Elongation at Break (%)
PLA	2810 ± 40	60 ± 2	3.7 ± 0.6
OFI10	2760 ± 80	54 ± 2	2.4 ± 0.5
OFI20	2610 ± 120	32 ± 5	1.8 ± 0.4
POL10	2780 ± 53	52 ± 2	3.4 ± 0.2
POL20	2560 ± 72	38 ± 7	2.3 ± 0.5

**Table 4:** Flexural properties of the 3D printed samples.

Sample	Flexural Modulus (MPa)	Flexural Strength (MPa)
PLA	550 ± 11	98 ± 6
OFI10	450 ± 17	58 ± 4
OFI20	353 ± 22	46 ± 2
POL10	485 ± 14	76 ± 8
POL20	350 ± 10	60 ± 4

**Table 5:** Impact properties of the 3D printed samples.

Sample	Impact Strength (kJ/m^2^)
PLA	21.2 ± 0.5
OFI10	25.4 ± 0.7
OFI20	11.3 ± 0.4
POL10	23.9 ± 1
POL20	15.9 ± 0.8

**Table 6:** Relative variations of the main mechanical properties in the biocomposites.

Sample	E	TS	EB	FM	FS	IS
OFI10	−1.8%	−10%	−35%	−18%	−41%	+20%
OFI20	−7%	−47%	−51%	−36%	−53%	−47%
POL10	−1%	−10%	−8%	−11%	−22%	+14%
POL20	−9%	−37%	−37%	−36%	−38%	−28%

**Table 7:** Molecular weights, expressed in kDa, of extruded (single-screw extrusion) and FDM samples.

Sample	Extruded	FDM
PLA	101.5	80.4
OFI10	99.7	76.0
OFI20	81.8	74.1
POL10	98.1	78.8
POL20	80.9	76.1

**Table 8:** WCA mean values and standard deviations collected from measurements on surfaces of FDM samples.

Sample	WCA (°)
PLA	43.4 ± 0.2
OFI10	38.1 ± 0.1
OFI20	36.9 ± 0.2
POL10	39 ± 0.1
POL20	37.9 ± 0.1

## Data Availability

The data presented in this study are available on request from the corresponding authors.
